# A transdiagnostic network analysis of psychosocial-clinical-cognitive functioning in young people with bipolar and major depressive disorders

**DOI:** 10.3389/fpsyt.2026.1748315

**Published:** 2026-03-17

**Authors:** Longbin Du, Xiaofen Zong, Jinxin He, Mengyao Feng, Hongjie Li, Yupan Tan, Li Dong, Xia Sun, Yuanyuan Zhang, Shuxian Yin, Huan Peng, Jie Yao, Qi Wen, Maolin Hu

**Affiliations:** 1 Department of Psychiatry, Renmin Hospital of Wuhan University, Wuhan, Hubei, China; 2 Department of Psychiatry, Xiaogan Mental Health Center, Xiaogan, Hubei, China

**Keywords:** bipolar disorder, cognitive function, major depressive disorder, network analysis, psychopathology, young people

## Abstract

**Background:**

High rates of diagnostic conversion and comorbidity between bipolar disorder (BD) and major depressive disorder (MDD) necessitate a transdiagnostic approach to uncover shared mechanisms. Network analysis can model the complex interrelationships among clinical, psychosocial, and cognitive domains, which remain underexplored in an integrated manner.

**Method:**

This study included 1,332 participants aged 10-24 (689 patients with BD-I, BD-II, or MDD, and 643 healthy controls). All underwent comprehensive assessments for clinical symptoms, psychosocial factors, and cognitive performance. We employed exploratory graph analysis to identify network clusters, estimated centrality and bridge centrality to identify key nodes, and used the Network Comparison Test across cognitive subgroups derived from hierarchical clustering.

**Results:**

A transdiagnostic two-cluster structure was identified: a symptom-psychosocial cluster and a neurocognition cluster. Depression and anhedonia were the central nodes within the symptom-psychosocial cluster, while processing speed and attention were central in the neurocognition cluster. Attention and self-harm were the key bridge nodes connecting the two clusters. Cognitive stratification revealed higher nodal strength (visual learning, processing speed) and global strength in the low-cognitive subgroup.

**Conclusion:**

This study delineates a transdiagnostic network architecture in young people’s mood disorders, identifying critical central and bridge nodes as intervention targets. The findings advocate for a dimensional, cognitive-informed approach to understanding and treating BD and MDD.

## Introduction

There is growing recognition that psychiatric diagnostic categories are pragmatic, human-made constructs designed primarily to guide clinical communication, rather than precise representations of distinct underlying disease entities (1, 2). This fundamental limitation is particularly salient in the realm of mood disorders, as exemplified by the challenges in differentiating bipolar disorder (BD) from major depressive disorder (MDD). The high rates of diagnostic conversion, comorbidity, and substantial heterogeneity within these conditions underscore the potential inadequacy of rigid categorical boundaries (1, 3). Accumulating evidence further reveals considerable overlaps between BD and MDD across multiple domains, including clinical symptoms, cognitive profiles, neurobiology, and genetic vulnerabilities (4, 5). These commonalities strongly suggest that critical psychopathological processes transcend the current diagnostic divisions, necessitating a shift toward a transdiagnostic approach. Such an approach aims to identify shared mechanisms and therapeutic targets by focusing on dimensional constructs that cut across the traditional BD-MDD dichotomy.

Psychopathology in BD and MDD extends beyond core mood symptoms to encompass other critical dimensions, most notably cognitive deficits. Impairments in domains such as memory, executive function, processing speed, and attention are frequently observed in both disorders (6, 7). The severity of cognitive deficits often correlates with the intensity of clinical symptoms (8), implying a potential causal relationship between psychopathology and cognitive dysfunction that may inform prognostic predictions. Furthermore, given the heterogeneity of these disorders, patients can be stratified into distinct cognitive subgroups, each exhibiting characteristic profiles (9). Statistical clustering approaches offer a powerful alternative to traditional group-average methods for defining patient subtypes based on overall cognitive performance, as they are specifically designed to account for, rather than obscure, cognitive heterogeneity (10). Such stratification enables a more nuanced analysis of symptom-cognition relationships across different patient subtypes. External psychosocial factors, such as interpersonal relationships and social support, also play a critical role, frequently interacting with both psychiatric symptoms and cognitive performance throughout the illness course (11). In summary, clinical symptoms, cognitive function, and psychosocial factors demonstrate interrelated alterations, forming a complex system of interactions. Elucidating these dynamic relationships may provide valuable insights into disease mechanisms and facilitate personalized treatment strategies.

Given the intricate interconnections among symptoms, psychosocial factors, and cognition, network analysis offers a powerful statistical framework for modeling these relationships (12). This approach conceptualizes psychopathology as a system of interacting elements, enabling the visualization and quantification of complex relationships. As an integral component of network analysis, exploratory graph analysis provides a data-driven dimensionality reduction technique that identifies clusters of variables based on the strength of their connections (13). This method also facilitates the identification of central nodes (highly influential variables within a cluster) and bridge nodes (variables that connect different clusters), which may represent key psychopathological mechanisms, potential intervention targets, and pathways for cross-domain influence (14, 15). Although previous studies have used network analysis to examine symptom-symptom and symptom-cognition relationships both within and across diagnostic categories (12, 16–18), transdiagnostic network studies that integrate clinical symptoms, psychosocial factors, and cognitive domains in mood disorders remain scarce.

Crucially, no study to date has specifically investigated the integrated psychosocial–symptom–cognitive network structure in young people with BD and MDD, despite the critical importance of this developmental period. Adolescence and early adulthood represent a peak window for the onset and progression of mood disorders, during which interpersonal relationships, social functioning, and cognitive abilities undergo significant maturation and are particularly vulnerable to disruption (19–22). Identifying the central and bridge elements in this unique population could provide insights into early disease mechanisms and inform targeted interventions.

The present study aims to elucidate the relationships among clinical symptoms, psychosocial factors, and cognitive domains using a transdiagnostic network approach in a large sample of young people with BD, MDD, and healthy controls. Our specific objectives were: (a) to map the network structure of clinical, psychosocial, and cognitive domains using exploratory graph analysis to identify underlying communities; (b) to determine central nodes influencing the overall network and bridge nodes connecting communities; and (c) to interpret network variations across empirically derived cognitive subtypes.

## Methods

### Participants

A total of 1,332 participants were included in this study: 261 patients with MDD, 199 with BD-I, 229 with BD-II, and 643 healthy controls (HCs). All participants were recruited between March 2024 and August 2025. Patient participants were identified from the inpatient psychiatry unit of Renmin Hospital of Wuhan University and were aged 10–24 years. Their diagnoses were confirmed by trained psychiatrists using the SCID-I/P. HCs were recruited during the same period from primary, junior high, and senior high schools in Wuhan and surrounding areas via advertisements. Key inclusion criteria for HCs included being aged 10–24 years, having no personal history of psychiatric disorders (as verified by the SCID Non-Patient Version), and having no family history of major psychiatric disorders.

For all participants, exclusion criteria consisted of significant physical illness, neurological disorders, history of substance abuse, or severe verbal/visual impairments.

The study received approval from the Institutional Review Board of Renmin Hospital, Wuhan University (Approval No. WDRY2024-K039), and written informed consent was obtained from all participants and their guardians.

### Assessment of clinical symptoms and psychosocial factors

Clinical symptoms and psychosocial factors were assessed using standardized instruments. Clinical symptoms included depression [evaluated with the Patient Health Questionnaire-9, PHQ-9 (23)], anxiety [assessed using the Generalized Anxiety Disorder-7, GAD-7 (24)], mania [screened by the Mood Disorder Questionnaire, MDQ (25)], anhedonia [measured with the Snaith–Hamilton Pleasure Scale, SHAPS (26)], insomnia [evaluated via the Insomnia Severity Index, ISI (27)], general psychiatric symptoms [assessed with the Brief Psychiatric Rating Scale, BPRS (28)], and non-suicidal self-harm (measured by the Chinese version of the Adolescent Non-Suicidal Self-Injury Questionnaire, NSSI; see **Supplementary Table 1**).

Psychosocial factors included interpersonal relationships (assessed using the Interpersonal Relationship Integrative Diagnostic Scale, IRIDS; **Supplementary Table 2**) and social support status [evaluated with the Social Support Rating Scale, SSRS (29)].

### Assessment of cognitive performance

Cognitive performance was assessed using the MATRICS Consensus Cognitive Battery–Chinese version (MCCB) (30, 31). The Chinese version of the MCCB has been validated in adolescents and young adults (32). The battery evaluates six neurocognitive domains and one social cognitive domain, which are detailed as follows:

- Processing Speed: Trail Making Test (TMT-A), Brief Assessment of Cognition in Schizophrenia (BACS), and Verbal Fluency Test (VFT);
- Attention: Continuous Performance Test (CPT);
- Working Memory: Wechsler Memory Scale Spatial Span (WMS-SS);
- Verbal Learning: Hopkins Verbal Learning Test (HVLT);
- Visual Learning: Brief Visuospatial Memory Test (BVMT);
- Reasoning: the Mazes subtest from the Neuropsychological Assessment Battery (NAB Mazes);
- Social Cognition: the Mayer–Salovey–Caruso Emotional Intelligence Test (MSCEIT).

We computed Z-scores for the nine MCCB subtests using the formula: Z-score = (raw score - Mean of HCs)/Standard Deviation of HCs. The cognitive domain scores were then derived by summing the Z-scores of the respective subtests within each domain (**Supplementary Table 3**).

Internal consistency was evaluated using Cronbach’s α. The results demonstrated acceptable internal consistency, with a Cronbach’s α of 0.714 for the total sample.

### Network analysis and centrality estimation in transdiagnostic samples

Network analysis was performed using R version 4.4.3. The primary transdiagnostic network was estimated using the combined patient sample (BD and MDD, N = 689) to capture shared psychopathological mechanisms. Healthy controls were excluded from this estimation to prevent spurious correlations driven by group differences. Each node represented the total score of a clinical symptom or psychosocial factor scale, and edges denoted partial correlations between nodes, with thickness reflecting connection strength. To enhance interpretability, we applied the EBICglasso model (γ = 0.5) to regularize the network by shrinking weak edges to zero, thereby emphasizing robust connections (33). The Fruchterman–Reingold algorithm was used for layout, positioning central nodes prominently (34). Nodal centrality was assessed using the R package qgraph (35), with strength as the primary metric for ranking.

To identify potential clusters of clinical symptoms, psychosocial factors and cognitive domains, we conducted exploratory graph analysis via the EGAnet package, implementing the walktrap algorithm to detect community structures (36, 37). Bridge centrality, particularly bridge strength, was computed to evaluate cross-community influences (15).

In addition, mediation analysis was performed to further investigate the causal pathway wherein key bridge nodes mediate the relationship from the symptom-psychosocial cluster to the neurocognition cluster (see method in **Supplementary Materials**).

### Hierarchical clustering analysis

Cluster analysis was conducted to identify cognitive subgroups across BDs and MDDs. First, the summed z-scores for each domain were themselves standardized. Subsequently, hierarchical clustering was applied using Euclidean distance and Ward’s linkage (38, 39). The optimal number of clusters (ranging from 2 to 6) was determined by evaluating a consensus of 30 indices provided by the “NbClust” R package (40).

### Network comparison test

The Network Comparison Test (NCT), a resampling-based permutation method, was used to examine differences in both global indices (global network and global strength invariance) and nodal indices (nodal strength and nodal bridge strength invariance) across cognitive clusters (41). Additionally, we assessed the potential effects of diagnosis, sex, and medication status on these four global and nodal metrics by comparing differences between diagnostic groups, gender groups, and three medication categories (drug-naïve, drug-free, and medicated groups).

## Results

### Sample characteristics

Relative to HCs, patients showed widespread significant impairments across clinical, psychosocial, and cognitive measures (all *P* < 0.001), apart from MCCB-Visual Learning (*P* = 0.382, **Table 1**). The groups (HCs, BD-I, BD-II, MDD) were well-matched on all demographic and clinical variables (all *P* > 0.05, **Table 1**).

**Table 1:** Demographic and clinical information in patient and control groups.

Clinical data	HCs n=643	BD-I n=199	BD-II n=229	MDD n=261	F/χ^2^	*P*
Age (years, M ± SD)	16.11 ± 2.66	16.42 ± 2.57	16.17 ± 2.55	16.04 ± 3.01	0.84	0.471
female/male	440/203	127/72	173/56	181/80	7.20	0.066
Education (years, M ± SD)	9.95 ± 2.47	9.83 ± 2.38	9.78 ± 2.55	9.69 ± 2.83	0.70	0.550
Disease Duration (months, M ± SD)	–	24.50 ± 21.75	27.28 ± 22.80	21.66 ± 21.90	3.85	0.022
Medication use^a^						
Drug naïve	–	85	112	158	1.50	0.221
Drug free	–	110	114	102		
Prior psychotropic drug use within the drug-free group						
Antipsychotics, no.	–	70	62	34		
Antidepressants, no.	–	58	74	74		
Mood stabilizers, no.	–	59	51	22		
Hypnotics, no.	–	10	16	17		
Cognitive functioning and psychopathology						
Processing Speed	0.00 ± 2.20	-2.50 ± 2.07	-2.21 ± 2.12	-2.32 ± 2.09	131.43	<0.001
Verbal Learning	0.00 ± 2.51	-0.37 ± 2.77	-0.10 ± 2.70	-0.06 ± 2.71	1.02	<0.001
Visual Learning	0.00 ± 2.55	-1.56 ± 3.71	-0.26 ± 3.08	-0.70 ± 3.31	14.84	0.382
Attention	0.00 ± 2.50	-2.18 ± 2.66	-1.59 ± 2.79	-1.75 ± 2.70	55.63	<0.001
Working Memory	0.00 ± 1.00	-0.51 ± 1.08	-0.27 ± 1.09	-0.27 ± 1.07	14.00	<0.001
Reasoning	0.00 ± 1.00	-0.63 ± 1.34	-0.53 ± 1.23	-0.44 ± 1.16	24.99	<0.001
Depression	5.64 ± 4.32	14.20 ± 7.58	17.60 ± 6.21	17.88 ± 5.62	467.12	<0.001
Anhedonia	21.74 ± 6.34	28.61 ± 8.12	32.73 ± 7.46	33.07 ± 7.82	232.44	<0.001
Anxiety	4.21 ± 3.62	10.58 ± 6.13	12.37 ± 5.17	12.57 ± 5.41	305.03	<0.001
Insomnia	6.52 ± 4.87	12.07 ± 7.56	13.76 ± 6.64	13.93 ± 6.47	147.87	<0.001
Psychotic Symptoms	19.28 ± 2.19	30.56 ± 7.27	32.02 ± 6.69	32.06 ± 6.90	617.99	<0.001
Mania	4.38 ± 2.58	6.68 ± 3.22	6.03 ± 3.13	4.41 ± 2.60	48.62	<0.001
Self-harm	4.20 ± 9.51	19.34 ± 19.93	26.57 ± 21.09	22.05 ± 19.71	156.02	<0.001
Interpersonal Relationship	8.53 ± 5.27	14.92 ± 7.13	17.20 ± 5.44	17.08 ± 5.31	230.14	<0.001
Social Support	0.03 ± 0.01	0.04 ± 0.01	0.04 ± 0.01	0.04 ± 0.01	127.19	<0.001
Social Cognition	0.00 ± 1.00	-0.48 ± 1.18	-0.82 ± 1.08	-0.87 ± 1.05	61.28	<0.001

HCs, Healthy controls; BD-I, Bipolar disorder type I; BD-II, Bipolar disorder type II; MDD, Major depressive disorder.

^a^Medication use data were missing for four patients with BD-I, three with BD-II, and one with MDD.

^b^“Drug naïve” refers to patients who have never been treated with psychotropic medications. “Drug free” refers to patients who were previously medicated but had discontinued all psychotropic drugs for at least 2 weeks (or 6 weeks for fluoxetine) prior to assessment. “Prior psychotropic drug use” was ascertained through clinical interviews and review of medical records.

### Network analysis in patients and HCs

**Figure 1:**
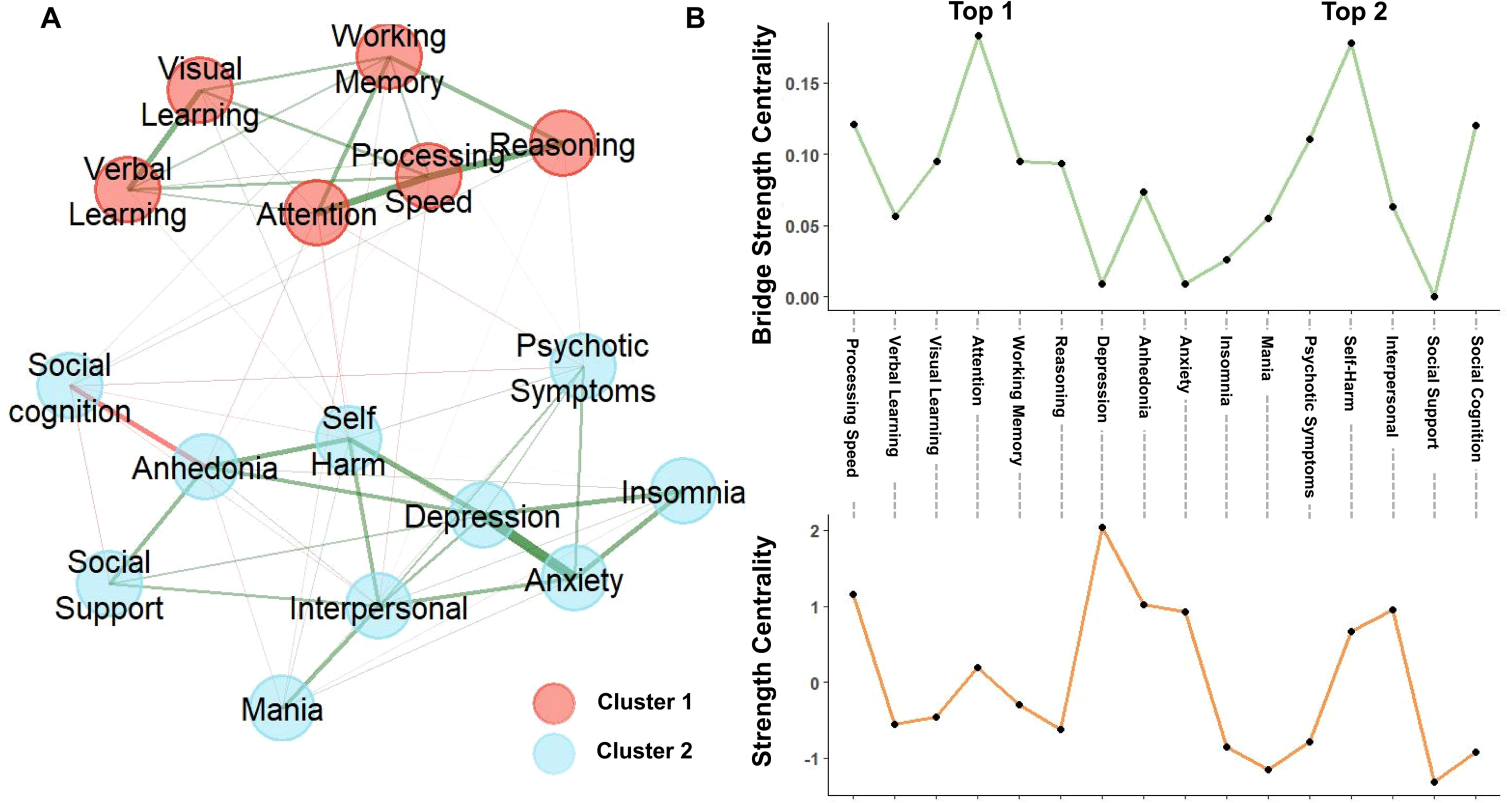
Transdiagnostic network of symptoms, psychosocial factors, and cognition in patients. **(A)** Exploratory graph analysis identified two robust clusters: one of clinical symptoms and psychosocial factors, and the other of neurocognitive domains. Green and red edges represent positive and negative correlations, respectively. **(B)** Nodal centrality. The bottom and top panels display strength centrality and bridge strength, respectively. The most central nodes in the entire network were depression and processing speed. Within the symptom-psychosocial and neurocognition clusters, the central nodes were depression/anhedonia and processing speed/attention, respectively. The key bridge nodes connecting both clusters were attention and self-harm.

We performed a network analysis with exploratory graph analysis on the entire patient group (BD and MDD), which identified two robust clusters (**Figure 1A**): a “symptom-psychosocial” cluster (comprising clinical symptoms, psychosocial factors, and social cognition) and a “neurocognition” cluster (consisting of neurocognitive domains). A similar clustering pattern was observed in HCs and across diagnostic subgroups (**Supplementary Figure 1**).

Next, we examined centrality indices within the overall network. Depression and Processing Speed emerged as the most central nodes based on their strength centrality (**Figure 1B**). Within the symptom-psychosocial cluster, depression and anhedonia showed the highest centrality; in the neurocognition cluster, processing speed and attention were the most central (**Figure 1B**). Bridge centrality analysis revealed that Attention and Self-harm had the highest bridge strength, underscoring their pivotal roles in connecting the two clusters (**Figure 1B**). Both nodal strength and bridge strength demonstrated acceptable stability (**Supplementary Figure 2**).

The NCT indicated no significant differences in global network properties (network invariance and global strength invariance) as well as nodal properties among these groups (all *P* > 0.05, **Supplementary Table 4**). Furthermore, nodes within the same cluster generally exhibited strong positive correlations (except for social cognition), whereas edges between clusters were predominantly negative and relatively weak (**Supplementary Figure 3**).

We found no significant effects of sex or medication use on global or nodal network metrics (all *P* > 0.05).

The results of structural equation model are presented in **Supplementary Materials** (**Supplementary Figure 4**).

### Network comparisons in cognitive subgroups

**Figure 2:**
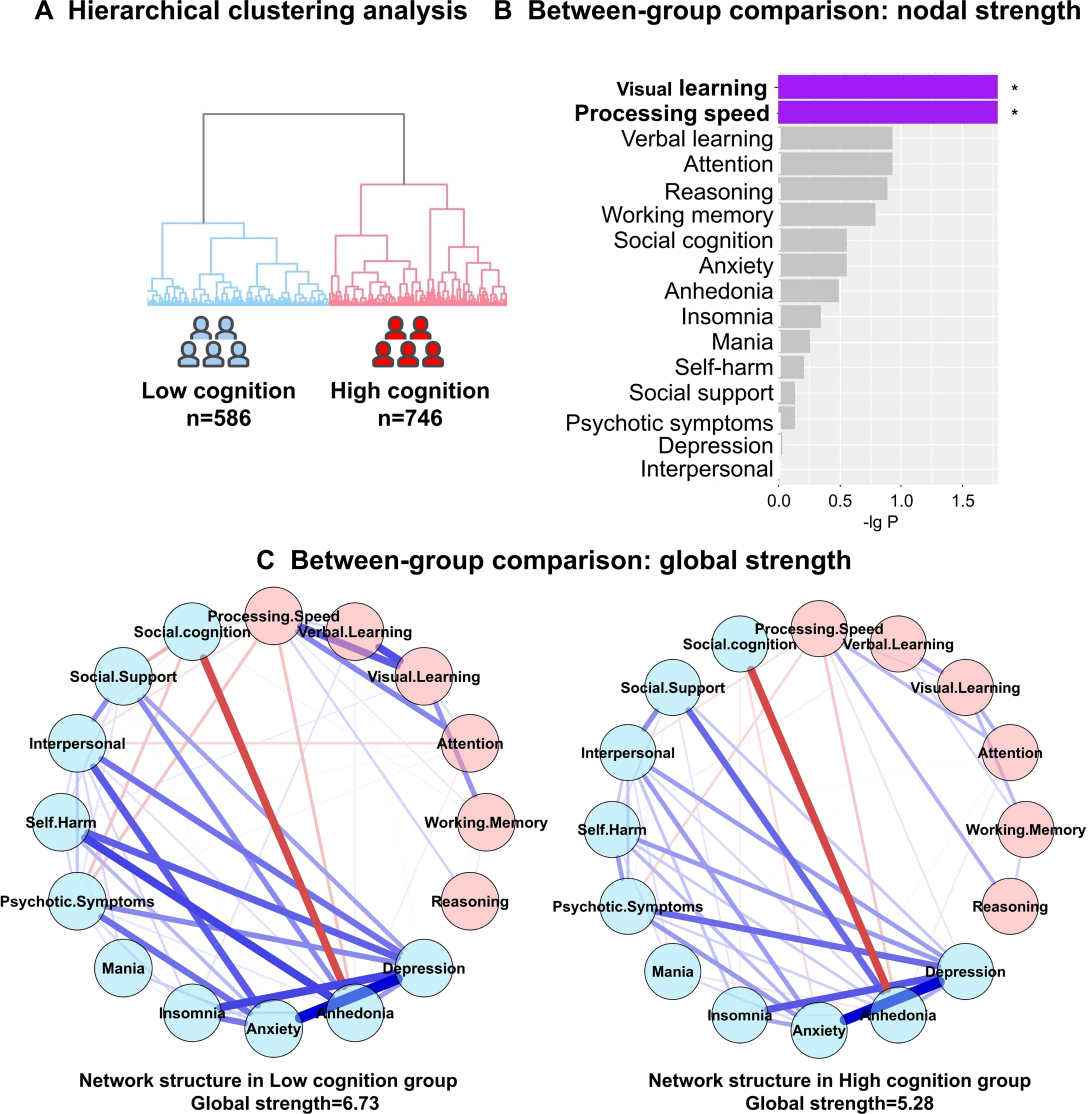
Transdiagnostic cluster analysis of cognitive performance and network comparison between cognitive subtypes. **(A)** Two cognitive subgroups identified by hierarchical clustering analysis; **(B)** Comparison of nodal strength between the two subgroups. Visual Learning and Processing Speed showed higher strength in the low-cognitive subgroup. The asterisk indicates a significant difference (*P* < 0.05) in nodal strength between the high- and low-cognition groups; **(C)** Comparison of global strength between the high- and low-cognition groups. The low-cognitive subgroup exhibited significantly greater global strength than the high-cognitive subgroup.

Hierarchical cluster analysis based on all thirty NbClust indices identified an optimal two-cluster solution, comprising high- and low-cognitive subgroups (**Figure 2A**). The high-cognitive subgroup performed significantly better across all cognitive domains than the low-cognitive subgroup (**Supplementary Table 5**). Nodal strength analysis further revealed that Visual Learning (*P* = 0.016) and Processing Speed (*P* = 0.016) exhibited higher strength in the low-cognitive subgroup (**Figure 2B**). However, no significant differences in the nodal bridge strength were detected between the two subgroups (*P* > 0.05).

Regarding global network properties, significant between-subgroup differences were found in network invariance (*P* = 0.015). In addition, global strength invariance was significantly greater in the low-cognitive subgroup than in the high-cognitive subgroup (*P* < 0.001; **Figure 2C**).

## Discussion

This study employed a transdiagnostic network approach to investigate the complex interrelationships among clinical symptoms, psychosocial factors, and cognitive domains in a large sample of young people with BD, MDD, and HCs, addressing a significant gap in the current literature. Our findings reveal several key insights. First, we identified a consistent, transdiagnostic network structure characterized by two robust clusters: a “symptom-psychosocial” cluster and a “neurocognition” cluster. Second, we identified central nodes within each cluster and key bridge nodes linking them, highlighting potential therapeutic targets and psychopathological pathways through which clinical states may influence cognitive function. Third, cognitive stratification showed that differences in global and nodal strength between high- and low-cognitive subgroups coexisted with stable cross-cluster connectivity, as indicated by invariant nodal bridge strength.

Our analysis identified a distinct dual-network architecture. This structure consists of a closely interrelated symptom-psychosocial cluster and a separate neurocognitive module. This separation suggests that mental states and psychosocial factors interact tightly to form a dynamic system. In contrast, cognitive function appears to be a more stable internal property that is less susceptible to short-term emotional or social fluctuations. This distinction aligns with previous evidence. While clinical symptoms tend to fluctuate with mood states, cognitive impairments often persist as trait-like markers even after acute episodes resolve (42–44).

The replication of this two-cluster pattern in HCs and across BD-I, BD-II, and MDD diagnostic subgroups is particularly noteworthy. It demonstrates that this psychopathological-cognitive organization is a fundamental characteristic of the population studied, rather than an artifact of illness. This finding aligns with both the previously proposed two-cluster pattern of “Psychopathology” and “Neurocognition” in transdiagnostic samples (12, 45) and the dimensional paradigms of the Hierarchical Taxonomy of Psychopathology (HiTOP) consortium (46) and the Research Domain Criteria (RDoC) framework (47). It thereby supports the view that core mental functioning operates along continua that cut across traditional diagnostic boundaries.

Within the symptom-psychosocial cluster, nodes were strongly interconnected, indicating synchronized variations among clinical and psychosocial variables and underscoring the multifactorial nature of mood disorders. Depression and anhedonia emerged as the most central nodes. Their prominence is consistent with their status as core features of both bipolar and depressive disorders, often exhibiting high comorbidity with anxiety, insomnia, and other symptoms (48–50). These central symptoms have been linked to increased risks of negative cognitions and sleep disturbances, reflecting the complex, bidirectional interactions within the psychopathology network (51). In the neurocognition cluster, processing speed and attention emerged as the most central nodes, with processing speed showing the highest strength centrality, suggesting it may play a particularly pivotal role. This aligns with previous studies proposing processing speed as a fundamental resource, whose impairment can mediate the relationship between depression and deficits in other cognitive domains like verbal, working memory, and visuo-spatial (52).

Bridge centrality analysis identified attention and self-harm as the pivotal connectors between the two clusters. This suggests a psychopathological pathway where individuals with self-harm behaviors are more likely to exhibit cognitive decline, particularly in the domain of attention. Self-harm is highly prevalent in severe psychiatric disorders among youth and has been historically associated with cognitive impairment (53–56). From the perspective of cognitive and affective neuroscience, this link may be driven by mechanisms such as emotional dysregulation and rumination. The cognitive effort required to regulate overwhelming negative affect likely competes for limited prefrontal resources, thereby depleting “top-down” attentional control (57). Furthermore, rumination-characterized by repetitive, passive focus on distress-acts as a persistent internal cognitive load. This internal interference may hijack attentional resources, making it difficult to disengage from negative stimuli and sustain attention on external tasks (58, 59). Our mediation analysis further substantiated this pathway, showing a significant indirect effect from the symptom-psychosocial cluster to neurocognition via self-harm and attention. However, this pathway accounted for only 20% of the total effect, underscoring the multifactorial and complex nature of cognitive impairment in mood disorders.

Our findings reflect the specific neurodevelopmental context of youth. The centrality of Attention and Processing Speed likely stems from the protracted maturation of the prefrontal cortex, which creates a window of vulnerability where emerging cognitive control systems are easily disrupted by emotional dysregulation (e.g., self-harm) (60). Theoretically, these functional links may be more fluid in youth compared to adults, where deficits often reflect structural “neurotoxic scars” from chronic illness (61). This implies that the youth network is more malleable, making interventions targeting the “Self-harm–Attention” pathway crucial for preventing the crystallization of cognitive deficits.

The stratification of patients into high- and low-cognitive subgroups revealed that network properties are not uniform across all individuals. We found that cognitive level influences global strength invariance and network invariance, whereas the nodal bridge strength remains relatively stable. Notably, the higher global strength observed in the low-cognitive subgroup suggests a more densely connected and rigid network configuration. According to the connectivity hypothesis in network theory, such strong interconnectivity often indicates greater vulnerability, where activation in one domain (e.g., depressive symptoms) can rapidly propagate to others (e.g., neurocognitive deficits), creating a self-sustaining feedback loop of impairment (62, 63). The specific enhancements in nodal strength for processing speed and visual learning in this subgroup indicate that these domains may act as critical “hubs” of dysfunction, driving the broader cognitive and clinical decline observed in these patients. Conversely, the lower global strength in the high-cognitive subgroup may reflect a state of network resilience. A less tightly coupled network implies that clinical symptoms are less likely to cascade into broad cognitive impairments, effectively buffering these individuals against severe functional decline despite the presence of mood disorders (64).

Despite these global differences, the stability of bridge strength across subgroups implies that the fundamental pathways linking psychopathology and cognition—specifically through Attention and Self-harm—are conserved mechanisms, operating independently of the patients’ overall cognitive baseline. This dissociation supports a two-tiered personalized intervention strategy. First, because bridge connections are stable, interventions targeting them (e.g., specific attention training or self-harm reduction programs) could serve as universal “circuit breakers”, beneficial across all cognitive profiles to prevent symptom-cognition cascading. Second, the higher network density in the low-cognitive subgroup necessitates stratified treatment intensity: these vulnerable patients may require intensive, multimodal interventions (e.g., combining pharmacotherapy with potent cognitive remediation targeting hubs like Processing Speed) to disrupt their rigid, self-sustaining networks, whereas less intensive preventive measures may suffice for the more resilient high-cognitive subgroup.

Several limitations of this study should be acknowledged. First, the cross-sectional design precludes conclusions regarding temporal dynamics or causal relationships among symptoms, psychosocial factors, and cognition. Longitudinal studies are needed to track network evolution over time. Second, although the sample was substantial, participants were recruited from a single center and restricted to the age range of 10–24 years, which may limit generalizability to other populations or age groups. Third, the reliance on self-report measures for clinical and psychosocial assessments introduces potential subjectivity. Finally, this study focused on behavioral and phenomenological data without incorporating neurobiological measures. Future research integrating multimodal data could help elucidate the brain-behavior mechanisms underlying the observed network patterns (65).

In summary, this study confirms the existence of transdiagnostic network structures linking psychopathology and neurocognition in young people with BD and MDD. We identified central nodes (depression, anhedonia, processing speed, attention) and critical bridge nodes (attention, self-harm) that may represent key targets for intervention. Furthermore, we demonstrated that network properties vary meaningfully across empirically derived cognitive subgroups, supporting a stratified approach to treatment. Our findings underscore the utility of the network perspective in untangling the complex interplay of symptoms, psychosocial factors, and cognition, offering a novel framework for understanding shared pathogenic pathways in young people’s mood disorders.

## Data Availability

The original contributions presented in the study are included in the article/**Supplementary Material**. Further inquiries can be directed to the corresponding author.

## References

[1] Hyman SE . The diagnosis of mental disorders: the problem of reification. Annu Rev Clin Psychol. (2010) 6:155–79. doi: 10.1146/annurev.clinpsy.3.022806.091532 17716032

[2] Marshall M . The hidden links between mental disorders. Nature. (2020) 581:19–21. doi: 10.1038/d41586-020-00922-8 32372044

[3] Olbert CM Gala GJ Tupler LA . Quantifying heterogeneity attributable to polythetic diagnostic criteria: theoretical framework and empirical application. J Abnorm Psychol. (2014) 123:452–62. doi: 10.1037/a0036068 24886017

[4] Gatt JM Burton KL Williams LM Schofield PR . Specific and common genes implicated across major mental disorders: a review of meta-analysis studies. J Psychiatr Res. (2015) 60:1–13. doi: 10.1016/j.jpsychires.2014.09.014 25287955

[5] Patel Y Parker N Shin J Howard D French L Thomopoulos SI . Virtual histology of cortical thickness and shared neurobiology in 6 psychiatric disorders. JAMA Psychiatry. (2021) 78:47–63. doi: 10.1001/jamapsychiatry.2020.2694 32857118 PMC7450410

[6] Martínez-Arán A Vieta E Colom F Reinares M Benabarre A Gastó C . Cognitive dysfunctions in bipolar disorder: evidence of neuropsychological disturbances. Psychother Psychosom. (2000) 69:2–18. doi: 10.1159/000012361 10601830

[7] Semkovska M Quinlivan L O'Grady T Johnson R Collins A O'Connor J . Cognitive function following a major depressive episode: a systematic review and meta-analysis. Lancet Psychiatry. (2019) 6:851–61. doi: 10.1016/S2215-0366(19)30291-3 31422920

[8] Pearson O Uglik-Marucha N Miskowiak KW Cairney SA Rosenzweig I Young AH . The relationship between sleep disturbance and cognitive impairment in mood disorders: a systematic review. J Affect Disord. (2023) 327:207–16. doi: 10.1016/j.jad.2023.01.114 36739007

[9] Iverson GL Brooks BL Langenecker SA Young AH . Identifying a cognitive impairment subgroup in adults with mood disorders. J Affect Disord. (2011) 132:360–7. doi: 10.1016/j.jad.2011.03.001 21439647 PMC4062916

[10] Green MJ Girshkin L Kremerskothen K Watkeys O Quidé Y . A systematic review of studies reporting data-driven cognitive subtypes across the psychosis spectrum. Neuropsychol Rev. (2020) 30:446–60. doi: 10.1007/s11065-019-09422-7 31853717

[11] Yang M Li J Fu Y Wang G Liu M Chen J . Association of childhood trauma, social support, cognition, and suicidality in females with bipolar disorder. BMC Psychiatry. (2024) 24:243. doi: 10.1186/s12888-024-05672-9 38566037 PMC10986031

[12] Chavez-Baldini U Nieman DH Keestra A Lok A Mocking RJT de Koning P . The relationship between cognitive functioning and psychopathology in patients with psychiatric disorders: a transdiagnostic network analysis. Psychol Med. (2023) 53:476–85. doi: 10.1017/S0033291721001781, PMID: 34165065 PMC9899564

[13] Hoffman M Steinley D Gates KM Prinstein MJ Brusco MJ . Detecting clusters/communities in social networks. Multivariate Behav Res. (2018) 53:57–73. doi: 10.1080/00273171.2017.1391682 29220584 PMC6103523

[14] Landherr A Friedl B Heidemann J . A critical review of centrality measures in social networks. Bus Inf Syst Eng. (2010) 2:371–85. doi: 10.1007/s12599-010-0127-3 41788969

[15] Jones PJ Ma R McNally RJ . Bridge centrality: a network approach to understanding comorbidity. Multivariate Behav Res. (2021) 56:353–67. doi: 10.1080/00273171.2019.1614898 31179765

[16] Zhao S Ji S Liu Y Han Y Wang A Fang W . Sex-specific networks of depressive symptoms and cognitive deficits in major depressive disorder: a network analysis approach. Gen Hosp Psych. (2025) 96:307–14. doi: 10.1016/j.genhosppsych.2025.08.009 40858057

[17] Park S-C Kim D . The centrality of depression and anxiety symptoms in major depressive disorder determined using a network analysis. J Affect Disord. (2020) 271:19–26. doi: 10.1016/j.jad.2020.03.078 32312693

[18] Zhu Y Zhang R Ni L Xie Z Lu S Xie S . The relationship between cognitive and global function in patients with schizophrenia and mood disorders: a transdiagnostic network analysis. Front Psychiatry. (2025) 16:1643369. doi: 10.3389/fpsyt.2025.1643369 40809864 PMC12344734

[19] Alloy LB Abramson LY Walshaw PD Keyser J Gerstein RK . A cognitive vulnerability–stress perspective on bipolar spectrum disorders in a normative adolescent brain, cognitive, and emotional development context. Dev Psychopathol. (2006) 18:1055–103. doi: 10.1017/S0954579406060524 17064429

[20] Suri D Teixeira CM Cagliostro MKC Mahadevia D Ansorge MS . Monoamine-sensitive developmental periods impacting adult emotional and cognitive behaviors. Neuropsychopharmacology. (2015) 40:88–112. doi: 10.1038/npp.2014.231 25178408 PMC4262911

[21] Ratheesh A Chen Y Hammond D Aitken Z Shah J Iorfino F . Progression of transdiagnostic stages from childhood to young adulthood. JAMA Psychiatry. (2025) 82:1113-22. doi: 10.1001/jamapsychiatry.2025.2648 40991272 PMC12461601

[22] Weintraub MJ Schneck CD Miklowitz DJ . Network analysis of mood symptoms in adolescents with or at high risk for bipolar disorder. Bipolar Disord. (2020) 22:128–38. doi: 10.1111/bdi.12870 31729789 PMC7085972

[23] Kroenke K Spitzer RL Williams JB . The PHQ-9: validity of a brief depression severity measure. J Gen Intern Med. (2001) 16:606–13. doi: 10.1046/j.1525-1497.2001.016009606.x 11556941 PMC1495268

[24] Spitzer RL Kroenke K Williams JB Löwe B . A brief measure for assessing generalized anxiety disorder: the GAD-7. Arch Intern Med. (2006) 166:1092–7. doi: 10.1001/archinte.166.10.1092 16717171

[25] Hirschfeld RM . The Mood Disorder Questionnaire: a simple, patient-rated screening instrument for bipolar disorder. Prim Care Companion J Clin Psychiatry. (2002) 4:9–11. doi: 10.4088/PCC.v04n0104 15014728 PMC314375

[26] Snaith RP Hamilton M Morley S Humayan A Hargreaves D Trigwell P . A scale for the assessment of hedonic tone the Snaith-Hamilton Pleasure Scale. Br J Psychiatry. (1995) 167:99–103. doi: 10.1192/bjp.167.1.99 7551619

[27] Bastien CH Vallières A Morin CM . Validation of the Insomnia Severity Index as an outcome measure for insomnia research. Sleep Med. (2001) 2:297–307. doi: 10.1016/S1389-9457(00)00065-4 11438246

[28] Overall JE Gorham DR . The brief psychiatric rating scale. Psychol Rep. (1962) 10:799–812. doi: 10.2466/pr0.1962.10.3.799 28575268

[29] Xiao S . Theoretical basis and research application of the Social Support Rating Scale. Clin J Psychiatry. (1994) 4:98–100.

[30] Lai S Zhong S Wang Y Zhang Y Xue Y Zhao H . The prevalence and characteristics of MCCB cognitive impairment in unmedicated patients with bipolar II depression and major depressive disorder. J Affect Disord. (2022) 310:369–76. doi: 10.1016/j.jad.2022.04.153 35504401

[31] Liang S Yu W Ma X Luo S Zhang J Sun X . Psychometric properties of the MATRICS Consensus Cognitive Battery (MCCB) in Chinese patients with major depressive disorder. J Affect Disord. (2020) 265:132–8. doi: 10.1016/j.jad.2020.01.052 32090734

[32] Tan Y Zong X Li H Feng M He J Sun X . Establishment of local norms and standardization of the MATRICS consensus cognitive battery in Chinese adolescents. Front Psychiatry. (2025) 16:1675079. doi: 10.3389/fpsyt.2025.1675079 41040941 PMC12483994

[33] Foygel R Drton M . Extended Bayesian information criteria for Gaussian graphical models. Adv Neural Inf Process Syst. (2010) 23.

[34] Fruchterman TMJR Reingold EM . Graph drawing by force-directed placement. Software: Pract Exp. (1991) 21:1129–64.

[35] Epskamp S Cramer AOJ Waldorp LJ Schmittmann VD Borsboom D . qgraph: network visualizations of relationships in psychometric data. J Stat Softw. (2012) 48:1–18. doi: 10.18637/jss.v048.i04

[36] Golino HF Epskamp S . Exploratory graph analysis: a new approach for estimating the number of dimensions in psychological research. PloS One. (2017) 12:e0174035. doi: 10.1371/journal.pone.0174035 28594839 PMC5465941

[37] Pons P Latapy M . Computing communities in large networks using random walks. J Graph Algorithms Appl. (2006) 10:191–218. doi: 10.7155/jgaa.00124 34651189

[38] Ward JH . Hierarchical grouping to optimize an objective function. J Am Stat Assoc. (1963) 58:236–44. doi: 10.1080/01621459.1963.10500845 41783271

[39] Miskowiak KW Kjærstad HL Lemvigh CK Ambrosen KS Thorvald MS Kessing LV . Neurocognitive subgroups among newly diagnosed patients with schizophrenia spectrum or bipolar disorders: a hierarchical cluster analysis. J Psychiatr Res. (2023) 163:278–89. doi: 10.1016/j.jpsychires.2023.05.025 37244066

[40] Charrad M Ghazzali N Boiteau V Niknafs A . NbClust: an R package for determining the relevant number of clusters in a data set. J Stat Softw. (2014) 61:1–36. doi: 10.18637/jss.v061.i06

[41] van Borkulo CD van Bork R Boschloo L Kossakowski JJ Tio P Schoevers RA . Comparing network structures on three aspects: a permutation test. Psychol Methods. (2023) 28:1273–85. doi: 10.1037/met0000476 35404628

[42] Robinson LJ Nicol Ferrier I . Evolution of cognitive impairment in bipolar disorder: a systematic review of cross‐sectional evidence. Bipolar Disord. (2006) 8:103–16. doi: 10.1111/j.1399-5618.2006.00277.x 16542180

[43] Roux P Raust A Cannavo A-S Aubin V Aouizerate B Azorin J-M . Associations between residual depressive symptoms, cognition, and functioning in patients with euthymic bipolar disorder: results from the FACE-BD cohort. Br J Psychiatry. (2017) 211:381–7. doi: 10.1192/bjp.bp.117.201335 29051175

[44] Bhardwaj A Wilkinson P Srivastava C Sharma M . Cognitive deficits in euthymic patients with recurrent depression. J Nervous Ment Dis. (2010) 198:513–5. doi: 10.1097/NMD.0b013e3181e4c5ba 20611055

[45] Zheng T Zheng X Xue B Xiao S Zhang C . A network analysis of depressive symptoms and cognitive performance in older adults with multimorbidity: a nationwide population-based study. J Affect Disord. (2025) 383:78–86. doi: 10.1016/j.jad.2025.04.122 40274116

[46] Kotov R Krueger RF Watson D Achenbach TM Althoff RR Bagby RM . The Hierarchical Taxonomy of Psychopathology (HiTOP): a dimensional alternative to traditional nosologies. J Abnorm Psychol. (2017) 126:454. doi: 10.1037/abn0000258 28333488

[47] Insel T Cuthbert B Garvey M Heinssen R Pine DS Quinn K . Research Domain Criteria (RDoC): toward a new classification framework for research on mental disorders. Am J Psychiat. (2010) 167:748–51. doi: 10.1176/appi.ajp.2010.09091379 20595427

[48] Dimick MK Hird MA Fiksenbaum LM Mitchell RH Goldstein BI . Severe anhedonia among adolescents with bipolar disorder is common and associated with increased psychiatric symptom burden. J Psychiatr Res. (2021) 134:200–7. doi: 10.1016/j.jpsychires.2020.12.031 33412423

[49] Wu C Mu Q Gao W Lu S . The characteristics of anhedonia in depression: a review from a clinically oriented perspective. Transl Psychiatr. (2025) 15:90. doi: 10.1038/s41398-025-03310-w 40118858 PMC11928558

[50] Whitton AE Pizzagalli DA . Anhedonia in depression and bipolar disorder. In: Pizzagalli DA , editor. Anhedonia: Preclinical, Translational, and Clinical Integration. Springer International Publishing, Cham (2022). p. 111–27. 10.1007/7854_2022_323 PMC12665610 35397065

[51] Barthel AL Pinaire MA Curtiss JE Baker AW Brown ML Hoeppner SS . Anhedonia is central for the association between quality of life, metacognition, sleep, and affective symptoms in generalized anxiety disorder: A complex network analysis. J Affect Disord. (2020) 277:1013–21. doi: 10.1016/j.jad.2020.08.077 33065810 PMC7575821

[52] Zaremba D Kalthoff IS Förster K Redlich R Grotegerd D Leehr EJ . The effects of processing speed on memory impairment in patients with major depressive disorder. Prog Neuro-Psychopharmacol Biol Psychiatry. (2019) 92:494–500. doi: 10.1016/j.pnpbp.2019.02.015 30831198

[53] Lu X Zhang Y Zhong S Lai S Yan S Song X . Cognitive impairment in major depressive disorder with non-suicidal self-injury: association with the functional connectivity of frontotemporal cortex. J Psychiatr Res. (2024) 177:219–27. doi: 10.1016/j.jpsychires.2024.07.008 39033667

[54] Turner BJ Austin SB Chapman AL . Treating nonsuicidal self-injury: a systematic review of psychological and pharmacological interventions. Can J Psychiatry. (2014) 59:576–85. doi: 10.1177/070674371405901103 25565473 PMC4244876

[55] Hu Z Han Y Hu M Zhang H Yuan X Yu H . A comparative study of cognitive function in young patients with bipolar disorder with and without non-suicidal self-injury. Acta Psychol. (2024) 243:104137. doi: 10.1016/j.actpsy.2024.104137 38228072

[56] Yin Q Xu H Chen Z Jiang Q Liu T . Detection of suicide risk using event-related potentials: a comprehensive systematic review and meta-analysis. Psychoradiology. (2025) 5:kkaf018. doi: 10.1093/psyrad/kkaf018 40586054 PMC12205307

[57] Ochsner KN Gross JJ . The cognitive control of emotion. Trends Cognit Sci. (2005) 9:242–9. doi: 10.1016/j.tics.2005.03.010 15866151

[58] Tonta KE Howell JA Hasking PA Boyes ME Clarke PJ . Attention biases in perfectionism: Biased disengagement of attention from emotionally negative stimuli. J Behav Ther Exp Psychiatry. (2019) 64:72–9. doi: 10.1016/j.jbtep.2019.02.009 30852359

[59] Whitmer AJ Gotlib IH . An attentional scope model of rumination. Psychol Bull. (2013) 139:1036. doi: 10.1037/a0030923 23244316 PMC3773498

[60] Casey BJ Getz S Galvan A . The adolescent brain. Dev Rev. (2008) 28:62–77. doi: 10.1016/j.dr.2007.08.003 18688292 PMC2500212

[61] Berk M Kapczinski F Andreazza AC Dean OM Giorlando F Maes M . Pathways underlying neuroprogression in bipolar disorder: focus on inflammation, oxidative stress and neurotrophic factors. Neurosci Biobehav Rev. (2011) 35:804–17. doi: 10.1016/j.neubiorev.2010.10.001 20934453

[62] Borsboom D . A network theory of mental disorders. World Psychiatry. (2017) 16:5–13. doi: 10.1002/wps.20375 28127906 PMC5269502

[63] Wichers M Riese H Hodges TM Snippe E Bos FM . A narrative review of network studies in depression: What different methodological approaches tell us about depression. Front Psychiatry. (2021) 12:719490. doi: 10.3389/fpsyt.2021.719490 34777038 PMC8581034

[64] Ponsoni A Branco LD Cotrena C Shansis FM Fonseca RP . The effects of cognitive reserve and depressive symptoms on cognitive performance in major depression and bipolar disorder. J Affect Disord. (2020) 274:813–8. doi: 10.1016/j.jad.2020.05.143 32664019

[65] Zong X Zhang J Li L Yao T Ma S Kang L . Virtual histology of morphometric similarity network after risperidone monotherapy and imaging-epigenetic biomarkers for treatment response in first-episode schizophrenia. Asian J Psychiatr. (2023) 80:103406. doi: 10.1016/j.ajp.2022.103406 36586357

