# Supplementary material for: A transdiagnostic network analysis of psychosocial-clinical-cognitive functioning in young people with bipolar and major depressive disorders
Source: Front Psychiatry. 2026 Mar 17;17:1748315. doi: 10.3389/fpsyt.2026.1748315 (PMC13036140; doi:10.3389/fpsyt.2026.1748315)
Supplement: Supplementary file 1 [file Table1.docx]

**A transdiagnostic network analysis of** **psychosocial-clinical-cognitive functioning in young people with** **bipolar and major depressive disorders**

Longbin Du^a,1^, Xiaofen Zong^a,1^, Jinxin He^a^, Mengyao Feng^a^, Hongjie Li^a^, Yupan Tan^a^, Li Dong^b^, Xia Sun^b^, Yuanyuan Zhang^b^, Shuxian Yin^b^, Huan Peng^b^, Jie Yao^b^, Qi Wen^b^, Maolin Hu^a,*^

^a^ Department of Psychiatry, Renmin Hospital of Wuhan University, Wuhan, Hubei, China

^b^ Department of Psychiatry, Xiaogan Mental Health Center, Xiaogan, Hubei, China

*Corresponding author at: Department of Psychiatry, Renmin Hospital of Wuhan University, Wuhan, Hubei, China

E-mail address: [humaolin@whu.edu.cn](mailto:humaolin@whu.edu.cn) (M.L. Hu)

^1^ These authors contributed equally to the work.

**Method**

Table S1. Description of Adolescent Non-suicidal Self-injury Questionnaire^[1]^

| Behavior in Your Past Life | Number of Occurrences (times) | | | |
| --- | --- | --- | --- | --- |
|  | 0 (0 point) | 1 (1 points) | 2-4 (2 points) | 5 (3 points) |
|  | The degree of harm to the body | | | |
|  | none (0 point) | mild (1 point) | moderate (2 point) | severe (3 point) |
| 1-Intentionally cut own skin with glass, knife, etc. | | | | |
| 2-Intentionally picked at wounds to prevent healing. | | | | |
| 3-Intentionally burned/scalded own skin with cigarettes, lighters, or other objects. | | | | |
| 4-Intentionally carved words or patterns on the body (excluding tattoos for aesthetic purposes). | | | | |
| 5-Intentionally scraped own skin until bleeding. | | | | |
| 6-Intentionally pierced skin or inserted objects under nails | | | | |
| 7-Intentionally banged head against objects, resulting in bruising. | | | | |
| 8-Intentionally pulled out own hair. | | | | |
| 9-Intentionally punched walls, glass, or other hard objects. | | | | |
| 10-Intentionally scratched oneself violently, causing scars or bleeding. | | | | |
| 11-Intentionally pierced body parts with needles, nails, or other objects to draw blood. | | | | |
| 12-Intentionally rubbed skin until bleeding. | | | | |
| 13-Intentionally hit oneself, resulting in bruising. | | | | |
| 14-Intentionally tied ropes or other objects around wrists or other body parts to constrict. | | | | |
| 15-Intentionally allowed others to hit or bite oneself to cause bodily harm. | | | | |
| 16-Intentionally exposed oneself to electric shock in non-life-threatening situations. | | | | |
| 17-Intentionally bit oneself until the skin broke. | | | | |
| 18-Intentionally set fire to hands or touched flames. | | | | |

Note: Scale instructions: Have you ever deliberately (not accidentally or unintentionally) engaged in any of the following behaviors without intending to end your life? How to fill out: Based on the description of "behaviors that have occurred in your past life," if the behavior objectively occurred, please indicate the approximate number of times it happened (0 times, 1 time, 2-4 times, 5 or more times). Then, indicate the degree of physical harm caused by this behavior (None, Mild, Moderate, Severe). Here, "None" means no damage to the skin, and "Severe" refers to a level of physical harm that requires hospitalization.

The score for each item is calculated by multiplying its "Number of Occurrences" score by its "The degree of harm to the body" score. The total score of the scale is the sum of the scores from all 18 items.

Table S2. Description of Interpersonal Relationship Integrative Diagnostic Scale^[2]^

| Item description | Options and Scores | |
| --- | --- | --- |
|  | Yes: 1 point | No: 0 point |
| 1-Difficulty expressing personal troubles | | |
| 2-Feeling uneasy when meeting strangers | | |
| 3-Excessive envy and jealousy toward others | | |
| 4-Insufficient interaction with the opposite sex | | |
| 5-Difficulty engaging in continuous conversation | | |
| 6-Feeling nervous in social situations | | |
| 7-Often hurting others | | |
| 8-Feeling uncomfortable interacting with the opposite sex | | |
| 9-Feeling lonely or lost even when surrounded by a large group of friends | | |
| 10-Easily embarrassed | | |
| 11-Inability to get along well with others | | |
| 12-Uncertainty about how to keep interactions with the opposite sex appropriate | | |
| 13-Feeling awkward when unfamiliar people share their life stories seeking sympathy | | |
| 14-Worrying about leaving a bad impression on others | | |
| 15-Always trying hard to gain others’ admiration | | |
| 16-Secretly admiring someone of the opposite sex | | |
| 17-Often avoiding expressing true feelings | | |
| 18-Lacking confidence in one’s appearance | | |
| 19-Disliking someone or being disliked by someone | | |
| 20-Looking down on the opposite sex | | |
| 21-Unable to listen attentively | | |
| 22-Having no one to confide in about personal troubles | | |
| 23-Feeling excluded or treated coldly by others | | |
| 24-Feeling looked down upon by the opposite sex | | |
| 25-Unable to consider diverse opinions or perspectives | | |
| 26-Often feeling hurt and sad in private | | |
| 27-Frequently being talked about or mocked by others | | |
| 28-Uncertainty about how to interact better with the opposite sex | | |

Note: IRIDS is a 28-question diagnostic scale to assess interpersonal behavioral distress, with each question answered “yes” (1 point) or “no”(0 point). **Score 0-8**: Demonstrates proficient social competence, characterized by sociability, initiative, and the maintenance of positive peer relationships. **Score 9-14**: Exhibits mild to moderate impairment in peer relations, evidenced by inconsistent and unstable friendships. **Score 15-28**: Indicates severe interpersonal dysfunction. A score exceeding 20 points to pronounced behavioral difficulties and potential psychopathology, including social isolation or maladaptive behaviors.

**Tabel.S3. Descriptions and calculation method of symptoms, psychosocial factors and neurocognition assessment**

| Measurement | Scales/Test | Calculation method |
| --- | --- | --- |
| Processing Speed | MCCB- Trail Making Test (TMT-A);  MCCB- The Brief Assessment of Cognition in Schizophrenia (BACS);  MCCB- Verbal Fluency Test (VFT) | Z (1/TMT-A) + Z (BACS)+ Z (Fluency) |
| Attention | MCCB- The Continuous Performance Test-Identical Pairs (CPT-IP) | Z (CPT-IP.1) + Z (CPT-IP.2) + Z (CPT-IP.3) |
| Working Memory | MCCB- The Wechsler Memory Scale (WMS-III): Spatial Span | Z (WMS-III) |
| Verbal Learning | MCCB- The Hopkins Verbal Learning Test-Revised™ (HVLT-R) | Z(HVLT-R.1) + Z(HVLT-R.2) + Z(HVLT-R.3) |
| Visual Learning | MCCB- The Brief Visuospatial Memory Test-Revised (BVMT-R) | Z(BVMT-R.1) + Z(BVMT-R.2) + Z(BVMT-R.3) |
| Reasoning | MCCB- Neuropsychological Assessment Battery (NAB®): Mazes (NAB-Mazes) | Z(NAB-Mazes) |
| Social Cognition | MCCB- Mayer–Salovey Emotional Intelligence Test (MSCEIT) | total score |
| Depression | Patient Health Questionnaire (PHQ-9) | total score |
| Anxiety | Generalized Anxiety Disorder Questionnaire (GAD-7) | total score |
| Mania | Mood Disorder Questionnaire, MDQ | total score |
| Anhedonia | Snaith-Hamilton-Pleasure scale (SHAPS) | total score |
| Insomnia | Insomnia Severity Index (ISI) | total score |
| Psychotic Symptoms | Brief Psychiatric Rating Scale (BPRS) | total score |
| Non-suicidal Self-harm | Adolescent Non-suicidal Self-injury Questionnaire (NSSI) | total score |
| Interpersonal Relationship | Interpersonal Relationship Integrative Diagnostic Scale (IRIDS) | total score |
| Social Support | Social Support Rating Scale (SSRS) | 1/total score |

Note: Z = z-score.

**Mediation analysis**

A mediation analysis was performed to investigate the causal pathway wherein key bridge nodes mediate the relationship from the symptom-psychosocial cluster to the neurocognition cluster. This was operationalized through a structural equation model (SEM) fitted with the R package “lavaan” [3]. The model specified the two clusters as latent variables and included the key bridge nodes as mediators. The model’s goodness-of-fit was evaluated against established benchmarks using the comparative fit index (CFI), the root-mean-square error of approximation (RMSEA), and the standardized root mean square residual (SRMR) [4].

**Results**

**Network analysis in patients and HCs**

**Fig. S1. Exploratory graph analysis in healthy controls and the three patient groups**

Results for (A) healthy controls, (B) bipolar disorder type I (BD-I), (C) bipolar disorder type II (BD-II), and (D) major depressive disorder (MDD) are shown. Nodes represent variables, color-coded by cluster. Green and red edges denote positive and negative correlations, respectively.

**Fig. S2. Stability of nodal strength and bridge strength**

The plot presents the stability of centrality indices assessed via case-dropping bootstrap. The x-axis indicates the proportion of omitted cases; the y-axis shows the average correlation between the centrality estimates from the subset and the original network. Lines trace the stability of strength and bridge strength, with shaded areas denoting the 95% confidence intervals.

**Table S4. Network comparison test (NCT) results across groups**

| Comparisons | Network Structure Invariance | | Global Strength Invariance | |
| --- | --- | --- | --- | --- |
|  | Test statistic M | *P-*values | Test statistic S | *P-*values |
| BD-Ⅰ vs. BD-Ⅱ | 0.150 | 0.798 | 0.591 | 0.535 |
| BD-Ⅰ vs. MDD | 0.161 | 0.602 | 0.257 | 0.782 |
| BD-Ⅰ vs. HCs | 0.151 | 0.784 | 0.839 | 0.725 |
| BD-Ⅱ vs. MDD | 0.142 | 0.852 | 0.333 | 0.737 |
| BD-Ⅱ vs. HCs | 0.162 | 0.523 | 0.248 | 0.887 |
| MDD vs. HCs | 0.189 | 0.247 | 0.582 | 0.754 |

Note: The Network Comparison Test (NCT) was performed with 1,000 permutations to assess the differences between groups. Test statistic M represents the test statistic for network structure invariance (the maximum difference in edge weights between the compared networks). Test statistic S represents the test statistic for global strength invariance (the absolute difference in global strength between the compared networks). BD-I: Bipolar Disorder Type I; BD-II: Bipolar Disorder Type II; MDD: Major Depressive Disorder; HCs: Healthy Controls.

**Fig. S3. Heatmap of the network's edge weights**

The plot represents the (A) the Pearson correlation matrix, (B) the thresholded adjacency matrix (sparsity threshold: gamma = 0.5). Positive and negative edge weights are represented by red and blue hues, respectively, as shown in the color scale. The cluster membership of each node is displayed along the left y-axis.

**Mediation analysis**

The results of the structural equation model are presented in Figure S4. Consistent with their central roles in the network analysis, “Depression” showed the strongest contribution to the “symptom-psychosocial” latent variable, whereas “processing speed” contributed most to the “neurocognition” latent variable. A significant direct effect was observed from the symptom-psychosocial factor to neurocognition (β= -0.065, 95% CI: -0.085 to -0.045, P < 0.001). Moreover, the indirect pathway through Self-harm and Attention was also statistically significant (β= -0.013, 95% CI: -0.021 to -0.005, P < 0.01), accounting for approximately 20% of the total effect. The model demonstrated good fit, as indicated by the following indices: CFI = 0.927, RMSEA = 0.071, SRMR = 0.061 (Fig. S4).

**Fig. S4.** **Structural equation model of the associations between clusters and key bridge nodes.**

Note: This model examines the relationship between the symptom-psychosocial and neurocognition constructs, mediated by Self-harm and Attention. Latent constructs (symptom-psychosocial and neurocognition) are represented by ellipses, along with their indicator variables and factor loadings listed in columns. Mediating variables (Self-harm and Attention) are displayed in rectangles. Path coefficients are shown on the gray arrows. CFI: comparative fit index; RMSEA: root mean square error of approximation; SRMR: standardized root mean square residual. ** *P* < 0.01, *** *P* < 0.001.

**Tabel S5. Variable scores and t-test between two cognitive subgroups**

| Clinical data | Low neurocognition  n=586 | High neurocognition  n=746 | t/χ2 | *P* |
| --- | --- | --- | --- | --- |
| Age (years, M±SD) | 15.45±2.82 | 16.70±2.48 | -8.59 | <0.001 |
| female/male | 447/139 | 474/272 | 24.97 | <0.001 |
| Education (years, M±SD) | 8.98±2.57 | 10.53±2.31 | -11.55 | 0.001 |
| **Diagnosis** |  |  |  |  |
| BD-Ⅰ | 130 | 69 | 4.489 | 0.106 |
| BD-Ⅱ | 136 | 93 |  |  |
| MDD | 145 | 116 |  |  |
| **Medication use in patients** ^a^ |  |  |  |  |
| Drug naïve | 210 | 145 | 0.031 | 0.861 |
| Drug free | 195 | 131 |  |  |
| **Prior psychotropic drug use within the drug-free group** |  |  |  |  |
| Antipsychotics, no. | 94 | 61 |  |  |
| Antidepressants, no. | 110 | 83 |  |  |
| Mood stabilizers, no. | 70 | 55 |  |  |
| Hypnotics, no. | 23 | 16 |  |  |
| **Cognitive functioning and psychopathology** |  | | |  |
| Processing Speed | -2.97±1.71 | 0.18±2.00 | -31.02 | < 0.001 |
| Verbal Learning | -1.05±2.82 | 0.68±2.18 | -12.28 | < 0.001 |
| Visual Learning | -1.25±3.61 | 0.24±2.31 | -8.72 | < 0.001 |
| Attention | -2.82±2.36 | 0.53±2.10 | -26.99 | < 0.001 |
| Working Memory | -0.66±1.01 | 0.21±0.94 | -16.312 | < 0.001 |
| Reasoning | -1.01±1.22 | 0.31±0.69 | -23.416 | < 0.001 |
| Depression | 13.39±8.22 | 9.80±7.27 | 8.331 | < 0.001 |
| Anhedonia | 29.59±8.82 | 24.75±8.17 | 10.372 | < 0.001 |
| Anxiety | 9.57±6.48 | 7.13±5.60 | 7.223 | < 0.001 |
| Insomnia | 11.27±7.26 | 9.10±6.45 | 5.68 | < 0.001 |
| Psychotic Symptoms | 28.43±8.69 | 23.50±6.85 | 11.21 | < 0.001 |
| Mania | 5.36±3.01 | 4.74±2.85 | 3.84 | < 0.001 |
| Self-harm | 18.93±21.43 | 9.87±14.89 | 8.69 | < 0.001 |
| Interpersonal Relationship | 14.39±7.13 | 11.29±6.43 | 8.23 | < 0.001 |
| Social Support | 0.04±0.01 | 0.03±0.01 | 6.05 | < 0.001 |
| Social Cognition | 0.69±1.51 | 0.20±1.06 | -6.723 | < 0.001 |

Notes: HCs: Healthy controls; BD-Ⅰ: Bipolar disorder type Ⅰ; BD-Ⅱ: Bipolar disorder type Ⅱ; MDD: Major depressive disorder.

a: Medication use data were missing for four patients with BD-I, three with BD-II, and one with MDD.

b: "Drug naïve" refers to patients who have never been treated with psychotropic medications. "Drug free" refers to patients who were previously medicated but had discontinued all psychotropic drugs for at least 2 weeks (or 6 weeks for fluoxetine) prior to assessment. "Prior psychotropic drug use" was ascertained through clinical interviews and review of medical records.

**Reference:**

[1] Yu Feng. The relation of adolescents’ self-harm behaviors, individual emotion characteristics and family environment factors. Master’s Thesis. Institute of Political Science, Central China Normal University. May, 2008. Supervisor: Jiang Guangrong.

[2] Zheng, R., 1999. Psychological diagnosis of college students. Shandong Education Press.

[3] Rosseel Y. lavaan: An R Package for Structural Equation Modeling. J Stat Softw. 2012;48(2):1 - 36.

[4] Kline RB. Principles and practice of structural equation modeling, fourth ed. New York: The Guilford Press; 2016. xvii, 534-xvii, p.
